# Supplementary material for: Litter Size of Sheep (Ovis aries): Inbreeding Depression and Homozygous Regions
Source: Genes (Basel). 2021 Jan 18;12(1):109. doi: 10.3390/genes12010109 (PMC7831309; doi:10.3390/genes12010109)
Supplement: Supplementary file 1 [file genes-12-00109-s001.zip › Fig S1.docx]

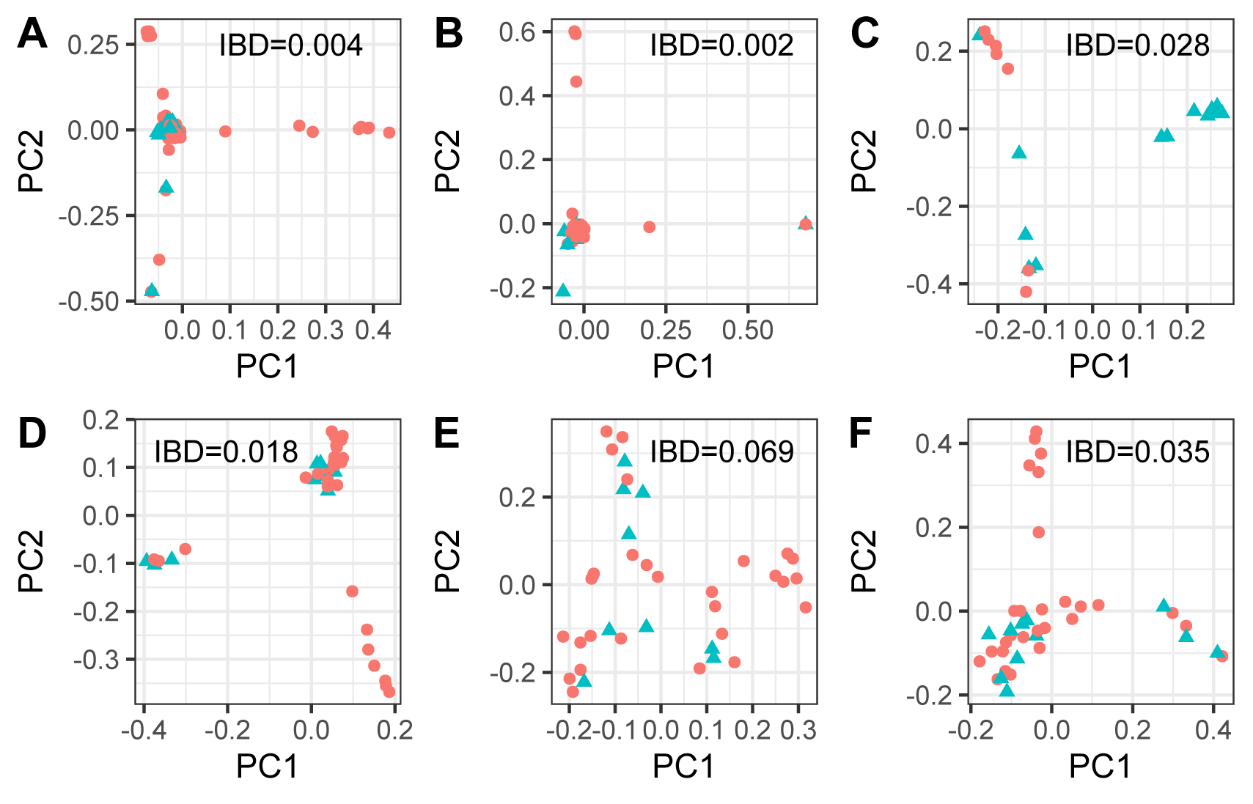
**Figure S1.** Principal component analysis of six breeds. A, Wadi; B, Hu; C, Icelandic; D, Finnsheep; E, Romanov; F, Texel. The blue triangle denotes the control, and the red solid circle denotes the case. The average pairwise identical by descent (IBD) was shown within each breed.
