# Supplementary material for: Litter Size of Sheep (Ovis aries): Inbreeding Depression and Homozygous Regions
Source: Genes (Basel). 2021 Jan 18;12(1):109. doi: 10.3390/genes12010109 (PMC7831309; doi:10.3390/genes12010109)
Supplement: Supplementary file 1 [file genes-12-00109-s001.zip › Fig S2.docx]

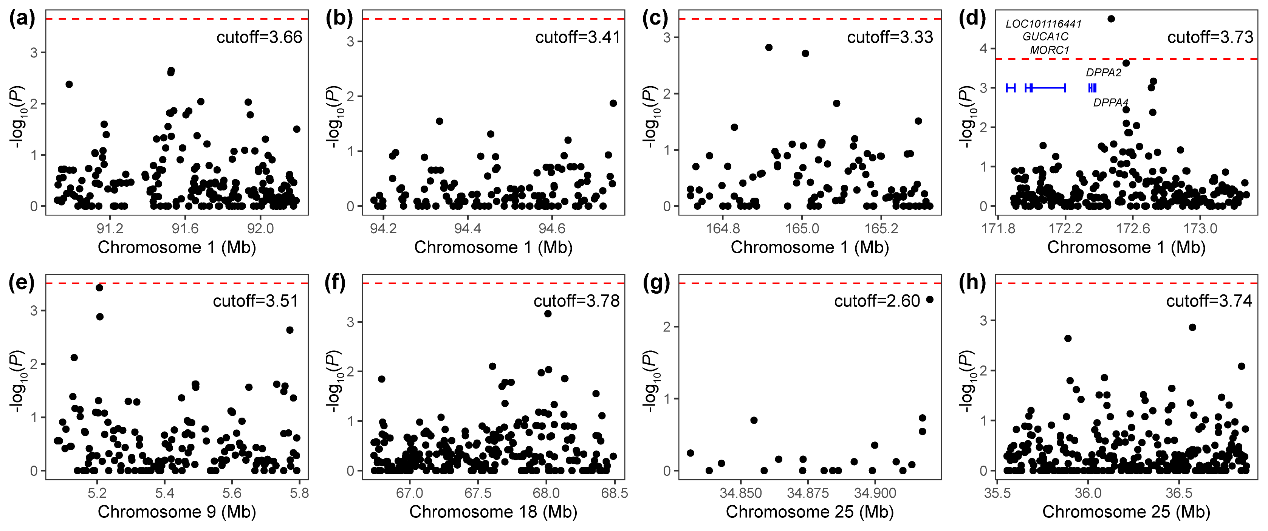


**Figure S2.** Regional Manhattan plots of candidate ROH hotspots. **a**, S311; **b**, S312; **c**, S125; **d**, S325; **e**, S378; **f**, S414; **g**, S736; **h**, S599. The red dotted lines indicate the cutoffs of Bonferroni corrections (0.05/the number of variations fell within each ROH hotspots). The blue line segments indicate the locations of candidate genes on chromosome 1.
