# Supplementary material for: Litter Size of Sheep (Ovis aries): Inbreeding Depression and Homozygous Regions
Source: Genes (Basel). 2021 Jan 18;12(1):109. doi: 10.3390/genes12010109 (PMC7831309; doi:10.3390/genes12010109)
Supplement: Supplementary file 1 [file genes-12-00109-s001.zip › Table S1.docx]

**Table S1.** Inbreeding coefficients based on ROH of six sheep breeds.

| **Length**  **(Mb)** | **F_ROH_ (mean ± SD, %)** | | | | | |
| --- | --- | --- | --- | --- | --- | --- |
|  | **Wadi** | **Hu** | **Icelandic** | **Finnsheep** | **Romanov** | **Texel** |
| 1-4 | 1.12±0.45 | 1.11±0.43 | 4.29±1.63 | 2.41±0.66 | 2.48±0.49 | 4.97±0.96 |
| 4-8 | 0.37±0.50 | 0.72±1.06 | 2.68±1.43 | 1.53±1.09 | 1.17±0.58 | 2.28±0.76 |
| > 8 | 1.71±4.76 | 7.51±13.55 | 4.59±5.04 | 2.59±3.80 | 1.78±1.06 | 2.47±1.27 |
| All | 3.20±5.29 | 9.34±14.65 | 11.56±6.61 | 6.53±4.97 | 4.83±1.56 | 9.72±1.80 |
